# Cytotoxicity of seputhecarpan D, thonningiol and 12 other phytochemicals from African flora towards human carcinoma cells

**DOI:** 10.1186/s12906-018-2109-9

**Published:** 2018-01-30

**Authors:** Victor Kuete, Dominique Ngnintedo, Ghislain W. Fotso, Oğuzhan Karaosmanoğlu, Bonaventure T. Ngadjui, Felix Keumedjio, Samuel O. Yeboah, Kerstin Andrae-Marobela, Hülya Sivas

**Affiliations:** 1 Department of Biochemistry, Faculty of Science, University of Dschang, Dschang, Cameroon; 2 Department of Biology, Science Faculty, Anadolu University, Eskişehir, Turkey; 3 Department of Organic Chemistry, Faculty of Science, University of Yaoundé I, Yaoundé, Cameroon; 4 Department of Biology, KamilÖzdağ Science Faculty, KaramanoğluMehmetbey University, Karaman, Turkey; 5 Department of Pharmacognosy and Pharmaceutical Sciences, Faculty of Medicine and Biomedical Science, University of Yaoundé I, Yaoundé, Cameroon; 6 Department of Chemistry, Faculty of Science, University of Botswana, Block 237, Private Bag 0022, Gaborone, Botswana; 7 Department of Biological Sciences, Faculty of Science, University of Botswana, Block 235, Private Bag, 0022, Gaborone, Botswana

**Keywords:** Africa, Carcinoma, Cytotoxicity, Mode of action, Seputhecarpan D, Thonningiol

## Abstract

**Background:**

Despite the remarkable progress in cancer therapy in recent years, this disease still remains a serious public health concern. The use of natural products has been and continues to be one of the most effective ways to fight malignancies. The cytotoxicity of 14 compounds from African medicinal plants was evaluated in four human carcinoma cell lines and normal fibroblasts. The tested samples included: *β*-spinasterol (**1**), friedelanone (**2**), 16*β*-hydroxylupeol (**3**), *β*-amyrin acetate (**4**), lupeol acetate (**5**), sequoyitol (**6**), rhamnitrin (**7**), europetin 3-*O*-rhamnoside (**8**), thonningiol (**9**), glyasperin F (**10**), seputhecarpan B (**11**), seputhecarpan C (**12**), seputhecarpan D (**13**) and rheediaxanthone A (**14**).

**Methods:**

The neutral red uptake (NR) assay was used to evaluate the cytotoxicity of samples; caspase-Glo assay, flow cytometry for cell cycle analysis and mitochondrial membrane potential (MMP) as well as spectrophotometry to measure levels of reactive oxygen species (ROS) were performed to detect the mode of action of compounds **9** and **13** in MCF-7 breast adenocarcinoma cells.

**Results:**

Compounds **3, 9–13** displayed cytotoxic effects against the four tested cancer cell lines with IC_50_ values below 85 μM. Compounds **9** and **13** had IC_50_ values below 10 μM in 4/4 and 3/4 tested cell lines respectively. The IC_50_ values varied from 0.36 μM (against MCF7 cells) to 5.65 μM (towards colon carcinoma DLD-1 cells) for **9**, from 9.78 μM (against MCF7 cells) to 67.68 μM (against HepG2 cells) for **13** and 0.18 μM (towards HepG2 cells) to 72 μM (towards Caco-2 cells) for the reference drug, doxorubicin. Compounds **9** and **13** induced cell cycle arrest in Go/G1 whilst doxorubicin induced arrest in G2/M. The two molecules (**9** and **13**) also induced apoptosis in MCF-7 cells through activation of caspases 3/7 and 9 as well as enhanced ROS production.

**Conclusion:**

Compounds **9** and **13** are good cytotoxic phytochemicals that should be explored more in future to develop a cytotoxic drug to fight human carcinoma.

**Electronic supplementary material:**

The online version of this article (10.1186/s12906-018-2109-9) contains supplementary material, which is available to authorized users.

## Background

Malignant diseases still constitute one of the major health problems worldwide, despite considerable progress in cancer therapy in recent years. Globally, about 14.1 million new cancer cases and 8.2 million deaths were reported in 2012 [1, 2], with lung, breast, colon, prostate and liver cancers being the most occurring neoplasia [3]. The mortality related to breast cancer reached 459,000 victims in 2008 [4] meanwhile colorectal cancer was reported as second killing cancer [5]. Every year, lung adenocarcinoma also kills more than one million persons and appears in top in cancer-related death [6]. The mortality assigned to liver cancer was 696,000 deaths in 2008 [7]. The fight against these types of cancers as well as many others is therefore of interest and should be intensified. The use of natural products has been and continues to be one of the most effective ways to fight cancers. Various compounds from African flora displayed prominent cytotoxic effects in vitro in many cancer cell lines [8, 9]. Within our drug discovery research program, the present study was planned to investigate the cytotoxicity of a panel of 14 natural products previously isolated from the Cameroonian medicinal plant *Garcinia epunctata* Stapf (Guttiferae) [10], *Ptycholobium contortum* (N.E.Br.) Brummitt (Leguminosae) from Botswana [11, 12], and freshly isolated from *Synsepalum zenkeri* Engl. ex Aubrév. & Pellegr. (Sapotaceae) harvested in Cameroon. The study was extended to the analysis of the mode of action of the two most active samples, namely the flavonoid thonningiol and the pterocarpan isoflavonoid, seputhecarpan D. Various flavonoids and pterocarpan isoflavonoids from African medicinal plants previously showed antiproliferative effects. Some prominent anticancer flavonoids of the flora of Africa previously identified in our cancer research program include isobavachalcone, 4-hydroxylonchocarpin [13, 14], 6,8-diprenyleriodictyol [13], cycloartocarpesin [14] and gancaonin Q [13] meanwhile examples of good cytotoxic pterocarpan isoflavonoids are sophorapterocarpan A and isoneorautenol [15, 16].

## Methods

### General procedure

NMR spectra were recorded on Bruker DMX Avance 300 and 400 instruments equipped with an auto-tune probe and using the automation mode aided by the Bruker program, Icon-NMR using Acetone-*d6*, CDCl_3_ and CD_3_OD as solvents and internal standards. HR EISMS spectra were determined on a microTOF-Q 98 spectrometer. Infra-Red spectra were recorded as KBr disk. For column chromatography, silica gel 60 particles size 0.04–0.063 mm (Merck) or sephadex LH-20 (Sigma) were used. Analytical and Preparative TLC were performed respectively using silica gel 60 PF_254 + 366_ (Merck) and silica gel 60-F_254_precoated aluminum sheets (Merck). The plates were visualized using UV (254 and 366 nm) and revealed by spraying with vanillin-sulphuric acid.

#### Chemicals

The control drug, doxorubicin (98.0%) was purchased from Sigma-Aldrich (Munich, Germany). The 14 tested compounds were: *β*-spinasterol (**1**), friedelanone (**2**), 16*β*-hydroxylupeol (**3**), *β*-amyrin acetate (**4**), lupeol acetate (**5**), 5-*O*-methyl-myo-inositol or sequoyitol (**6**), rhamnitrin or 7-*O*-methylquercetin 3-*O*-rhamnoside (**7**), europetin 3-*O*-rhamnoside or 7-*O*-methylmyricetin 3-*O*-rhamnoside (**8**), thonningiol (**9**), glyasperin F (**10**), seputhecarpan B (**11**), seputhecarpan C (**12**), seputhecarpan D (**13**) and rheediaxanthone A (**14**). The isolation and identification of compounds **2**, **3** and **14** from the Cameroonian medicinal plant *Garcinia epunctata* Stapf (Guttiferae) [10] and compounds **5, 9–13** from *Ptycholobium contortum* (N.E.Br.) Brummitt (Leguminosae) from Botswana [11, 12] were previously reported. *Garcinia epunctata* Stapf (Guttiferae) was collected in July 2011 at Eloumden inYaoundé (Centre Region of Cameroon) and identified by Mr. Victor Nana at the National Herbarium where a voucher specimen (19,534/SRFCam) was deposited [10]. *Ptycholobium contortum* was harvested around Maun, Ngamiland District in North-Western Botswana and identified by Joseph Madome of the Okavango Research Institute (ORI) Herbarium; voucher specimen (KM-2-Maun-2014) is available at at ORI Herbarium, respectively [11, 12]. Compounds **1, 4, 6, 7** and **8** were isolated from the bark, leaves and roots of *Synsepalum zenkeri* Engl. ex Aubrév. & Pellegr. (Sapotaceae) as described below.

### Plant material

*Synsepalum zenkeri* Engl. ex Aubrév. & Pellegr. (Sapotaceae) was collected from Mont Kalla, Yaoundé, Cameroon, in April 2014. The sample was authenticated by Mr. Nana Victor of the National Herbarium of Cameroon, Yaoundé, Cameroon under the voucher number 21816/SRFCAM.

### Extraction and isolation of compounds from *Synsepalum zenkeri*

Compounds isolated from leaves, bark and roots of *Synsepalum zenkeri* were *β*-amyrin acetate (**4**) [17], *β*-spinasterol (**1**) [18], rhamnitrin or 7-*O*-methylquercetin 3-*O*-rhamnoside (**7**) [19], europetin 3-*O*-rhamnoside or 7-*O*-methylmyricetin 3-*O*-rhamnoside (**8**) [19], and lupeol acetate **6** [20]. Details on extraction and their isolation are provided as (Additional file 1: S1).

#### Cell culture

Four human carcinoma cell lines and one normal cell line were used. They were: DLD-1 and Caco-2 colorectal adenocarcinoma cell lines, HepG2 hepatocarcinoma cells, MCF-7 breast adenocarcinoma cells and the normal CRL2120 human skin fibroblasts. Caco-2 cells (from the ŞAP Institute of Ankara,Turkey andMCF-7 cells (from ATCC) provided by Prof. Dr. Tansu Koparal (Anadolu University, Eskisehir, Turkey),HepG2, DLD-1 and CRL2120 cells were obtained from American Type Culture Collection (ATCC); DMEM medium (Sigma-aldrich, Munich, Germany) was used to maintain cells as a monolayer and was supplemented with 10% fetal calf serum and 1% penicillin (100 U/mL)-streptomycin (100 μg/mL) in a humidified 5% CO_2_ atmosphere at 37 °C.

#### Neutral red (NR) uptake assay

The cytotoxicity of compounds (**1–14**) and doxorubicin (positive control) was performed by NR uptake assay as previously described [21–24]. NR uptake assay is cheaper and more sensitive than other cytotoxicity tests and is based on the ability of viable cells to incorporate and bind the supravital dye NR in the lysosomes [25]. Dimethylsufoxide (DMSO) at less than 0.1% final concentration was used to dilute the tested samples. DMSO at 0.1% was used as solvent control. The incubation time was 72 h incubation in humidified 5% CO_2_ atmosphere at 37 °C, followed by NR coloration as previously described [23, 24]. ELx 808 Ultra Microplate Reader (Biotek) equipped with a 540 nm filter was used to measure the absorbance. Each experiment was performed at least three times, with three replicates each. The viability was evaluated based on a comparison with untreated cells. The IC_50_ values represented the sample’s concentrations required to inhibit 50% of cell proliferation and were calculated from a calibration curve by linear regression using Microsoft Excel [26] as follows: for each sample, the cell growth percentages at the different concentrations were used to draw the calibration curve with logarithmic regression obtained by 2 measuring points (one smaller and one larger than 50%) from where the IC_50_ value was deduced.

#### Cell cycle analysis and detection of apoptosis

The effect of compounds **9** and **13** and doxorubicin (positive control) in cell cycle distribution of MCF-7 cells was performed by flow cytometry using BD cycletest™ Plus DNA Kit Assay (BD Biosciences, San Jose, USA) as previously described [23]. Cells were tested in 6-well plates (3 mL, 1 × 10^5^ cells/mL) and the incubation time was 72 h in humidified 5% CO_2_ atmosphere at 37 °C. The tested concentrations were ¼ × IC_50_, ½ × IC_50_ and IC_50_. Untreated cells (control) were used for comparison with treated cells. The BD FACS Aria I Cell Sorter Flow Cytometer (Becton-Dickinson, Germany) was then used for cell cycle analysis. For each sample, 10^4^ cells were counted. For PI excitation, an argon-ion laser emitting at 488 nm was used. Cytographs were analyzed using BD FACSDiva™ Flow Cytometry Software Version 6.1.2 (Becton-Dickinson).

#### Caspase activity

Compounds **9** and **13** as well as doxorubicin (positive control) were used to treat MCF-7 cells for 6 h, followed by detection of caspase activity using Caspase-Glo 3/7 and Caspase-Glo 9 Assay kits (Promega, Mannheim, Germany) as previously reported [27–29]. Cells were incubated in humidified 5% CO_2_ atmosphere at 37 °C; the concentrations of samples used were ½ × IC_50_ and IC_50_ meanwhile DMSO was used as solvent control. The BioTek Synergy™ HT multi-detection microplate reader was used to measure the luminescence and Caspase activity was expressed as percentage of the untreated control.

#### Analysis of the integrity of mitochondrial membrane

The MCF-7 cells were treated with compounds **9** and **13** and doxorubicin, and the integrity of MMP was analyzed using 5,5′,6,6′-tetrachloro-1,1′,3,3′-tetraethylbenzimidazolylcarbocyanine iodide (JC-1; Biomol, Hamburg, Germany) staining as previously reported [27–29]. Cells were tested in 6-well plates (3 mL, 1 × 10^5^ cells/mL) and the incubation time was 72 h in humidified 5% CO_2_ atmosphere at 37 °C. The tested concentrations were ¼ × IC_50_, ½ × IC_50_ and IC_50_. Untreated cells (control) were used for comparison with treated cells. JC-1 staining was performed according to the manufacturer’s protocol as reported previously [23]. Cells were then measured in a BD FACS Aria I Cell Sorter Flow Cytometer (Becton-Dickinson, Germany). The JC-1 signal was measured at an excitation wavelength of 561 nm (150 mW) and detected using a 586/15 nm band-pass filter. The signal was analyzed at an excitation wavelength of 640 nm (40 mW) and detected using a 730/45 nm bandpass filter. Cytographs were analyzed using BD FACSDiva™ Flow Cytometry Software Version 6.1.2 (Becton-Dickinson). All experiments were performed at least in triplicates.

#### Measurement of reactive oxygen species production

The MCF-7 cells were treated with compounds **9** and **13** and doxorubicin, and ROS production was measured using 2′,7′-dichlorodihydrofluorescein diacetate (H_2_DCFH-DA) (Sigma-Aldrich) by OxiSelect™ Intracellular ROS Assay Kit (Green Fluorescence) as recommended by the manufacturer, Cell Biolabs Inc. (San Diego, USA) [23]. Cells were tested in 6-well plates (3 mL, 1 × 10^5^ cells/mL) and the incubation time was 24 h in humidified 5% CO_2_ atmosphere at 37 °C. The tested concentrations were ¼ × IC_50_, ½ × IC_50_ and IC_50_. Untreated cells (control) were used for comparison with treated cells. The fluorescence was measured using SpectraMax® M5 Microplate Reader (Molecular Devices, Biberach, Germany) at 480/530 nm. All experiments were performed at least in triplicates.

## Results

### Tested compounds

**Fig. 1:**
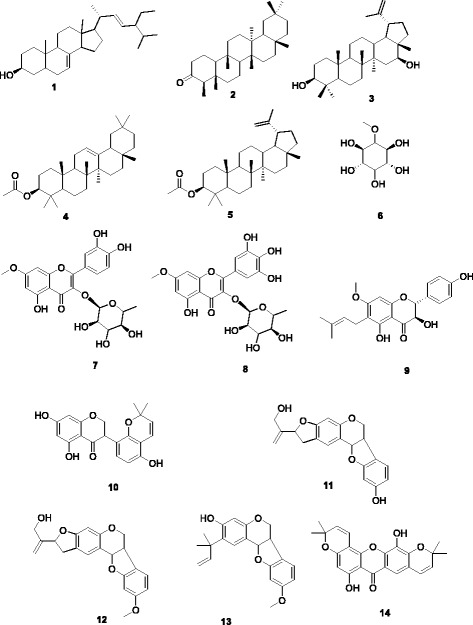
Chemical structures of tested compounds. 1: β-spinasterol; 2: friedelanone; 3: 16*β*-hydroxylupeol; 4: *β*-amyrin acetate; 5: 5-*O*-methyl-myo-inositol or sequoyitol; 6: lupeol acetate; 7: rhamnitrin or 7-*O*-methylquercetin 3-*O*-rhamnoside; 8: europetin 3-*O*-rhamnoside or 7-*O*-methylmyricetin 3-*O*-rhamnoside; 9: thonningiol; 10: glyasperin F; 11: seputhecarpan B; 12: seputhecarpan C; 13: seputhecarpan D; 14: rheediaxanthone A

Compounds tested (Fig. 1) were five terpenoids including one steroid: *β*-spinasterol (**1**) and 4 triterpenoids: friedelanone (**2**), 16*β*-hydroxylupeol (**3**), β-amyrin acetate (**4**) and lupeol acetate (**5**), one cyclitol: 5-*O*-methyl-myo-inositol or sequoyitol (**6**), 3 flavonoids: rhamnitrin or 7-*O*-methylquercetin 3-*O*-rhamnoside (**7**) and europetin 3-*O*-rhamnoside or 7-*O*-methylmyricetin 3-*O*-rhamnoside (**8**) and thonningiol (**9**); one isoflavonid: glyasperin F (**10**), three pterocarpan isoflavonoids: seputhecarpan B (**11**), seputhecarpan C C_21_H_20_O_5_ (**12**) and seputhecarpan D (**13**) and one xanthone: rheediaxanthone A (**14**). The isolation and identification of compounds **2**, **3** and **14** from *Garcinia epunctata* [10] and **5, 9–13** from *Ptycholobium contortum* [11, 12] were previously reported. Compounds **1, 4, 6, 7** and **8** were isolated from the bark, leaves and roots of *Synsepalum zenkeri* (Sapotaceae). Further details on the tested compounds are available in (Additional file 1: S2).

### Cytotoxicity

Compounds **3, 9–13** displayed antiproliferative effects against the four tested cancer cell lines with C_50_ values below 85 μM (Table 1). Other compounds showed selective cytotoxicity. The IC_50_ values varied from 9.12 μM (in MCF-7 breast adeconcarcinoma cells) to 35.64 μM (towards HepG2 hepatocarcinoma cells) for compound **3**, from 0.36 μM (against MCF-7 cells) to 5.65 μM (towards colon carcinoma DLD-1 cells) for **9**, from 11.14 μM (against MCF-7 cells) to 20.89 μM (towards DLD-1 colon carcinoma cells) for **10**, from 11.92 μM (against MCF-7 cells) to 84.41 μM (against Caco-2 colon carcinoma cells) for **11**, from 13.30 μM (in MCF-7 cells) to 76.72 μM (against HepG2 cells) for **12**, from 9.78 μM (against MCF-7 cells) to 67.68 μM (against HepG2 cells) for **13** and 0.18 μM (towards HepG2 cells) to 72 μM (towards Caco-2 cells) for doxorubicin. Compounds **1–14** were in all cases less toxic in normal CRL2120 fibroblasts than in cancer cells, with IC_50_ values above 45 μM (Table 1). Compounds **9** and **13** had IC_50_ values below 10 μM in 4/4 and 3/4 tested cell lines respectively.

**Table 1:** Cytotoxicity of tested compounds and doxorubicin in carcinoma and normal cell lines using NR assay

Compounds	Cell lines, IC_50_ values (in μM) and selectivity index^a^ (in bracket)				
	MCF7	DLD-1	Caco-2	HepG2	CRL2120
1	> 97.09	> 97.09	69.81 ± 5.12 (1.39)	> 97.09	> 97.09
2	70.82 ± 4.95 (> 1.33)	> 93.90	> 93.90	> 93.90	> 93.90
3	9.12 ± 1.02 (5.76)	15.41 ± 0.96 (3.41)	20.76 ± 1.53 (2.53)	35.64 ± 3.29 (1.47)	52.55 ± 5.11
4	> 85.47	> 85.47	> 85.47	> 85.47	> 85.47
5	10.68 ± 0.75 (> 8.00)	> 85.47	> 85.47	> 85.47	> 85.47
6	166.52 ± 12.04 (> 1.24)	> 206.19	> 206.19	45.10 ± 3.42 (> 4.57)	> 206.19
7	60.91 ± 5.39 (0.86)	> 86.58	> 86.58	> 86.58	52.27
8	16.74 ± 1.81 (> 5.00)	> 83.68	> 83.68	> 83.68	> 83.68
9	**0.36 ± 0.01 (124.59)**	**5.65 ± 0.03 (8.05)**	**5.28 ± 0.06 (8.60)**	**3.88 ± 0.04 (11.72)**	45.46 ± 4.17
10	11.14 ± 0.88 (> 10.14)	20.89 ± 1.64 (> 5.41)	17.60 ± 1.39 (> 6.42)	16.89 ± 1.20 (> 6.69)	> 112.99
11	11.92 ± 0.94 (9.93)	57.93 ± 3.89 (> 2.04)	84.41 ± 6.32 (> 1.40)	61.92 ± 4.20 (> 1.91)	> 118.34
12	13.30 ± 0.67 (> 8.55)	37.84 ± 1.76 (> 3.00)	67.29 ± 3.98 (> 1.69)	76.72 ± 7.24 (> 1.48)	> 113.64
13	**9.78 ± 0.55 (> 12.10)**	**10.78 ± 1.11 (> 10.97)**	**10.46 ± 0.48 (> 11.32)**	67.68 ± 5.18 (> 1.17)	> 118.34
14	> 102.04	> 102.04	**3.15 ± 0.01** (35.49)	14.60 ± 0.93 (5.50)	80.29 ± 5.42
Doxorubicin	**0.35 ± 0.05** (1.69)	**0.37 ± 0.05** (1.59)	**0.72 ± 0.13** (0.82)	**0.18 ± 0.03** (3.21)	**0.59 ± 0.01**

(^a^): The selectivity index was determined as the ratio of IC_50_ value in the CRL2120 normal fibroblasts divided by the IC_50_ in the cancer cell lines. 1: β-spinasterol; 2: friedelanone; 3: 16*β*-hydroxylupeol; 4: *β*-amyrin acetate; 5: sequoyitol; 6: lupeol acetate; h: rhamnitrin; 8: europetin 3-*O*-rhamnoside; 9: thonningiol; 10: glyasperin F; 11: seputhecarpan B; 12: seputhecarpan C; 13: seputhecarpan D; 14: rheediaxanthone A; In bold: significant activity [8, 30, 31, 41]

### Cell cycle distribution and modes of action

**Fig. 2:**
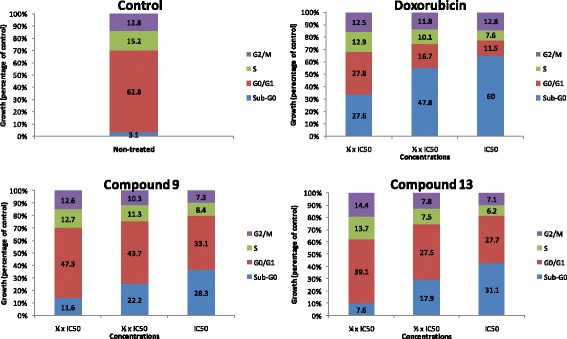
Cell cycle distribution of MCF-7 cells treated with thonningiol (9), seputhecarpan D (13) and doxorubicin for 72 h. IC_50_: 0.36 μM (thonningiol), 9.78 μM (seputhecarpan D) and 0.35 μM (doxorubicin)

The MCF-7 cells’ cycle distribution after treatments with compound **9** and **13** and doxorubicin was analyzed, and the results are depicted in Fig. 2. The two phytochemicals as well as doxorubicin altered the distribution of various phases of cell cycle in MCF-7 cells, with pronounced concentration-dependent increase of cells in sub-G0/G1 phase. Compounds **9** and **13** induced cell cycle arrest in Go/G1 whilst the reference molecule, doxorubicin induced arrest in G2/M. As indication of apoptosis, percentages of cells in sub-G0/G1 phase were in the ranges of 11.6% (¼ × IC_50_) to 28.3% (2 × IC_50_) for **9**, 7.6% (¼ × IC_50_) to 31.1% (2 × IC_50_) for **13**, and from 27.6% (¼ × IC_50_) to 60% (2 × IC_50_) for the reference drug, doxorubicin. The percentage of cells in sub-G0/G1 phase in non-treated cells was 3.1%.

**Fig. 3:**
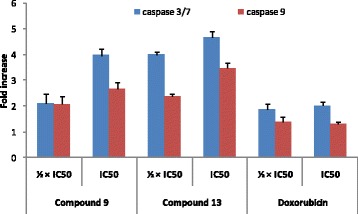
Activity of caspases 3/7 and 9 in MCF-7 cells treated with thonningiol (9), seputhecarpan D (13) and doxorubicin for 6 h. IC_50_: 0.36 μM (thonningiol), 9.78 μM (seputhecarpan D) and 0.35 μM (doxorubicin)

The activity of caspases in MCF-7 cells treated with compounds **9, 13** and doxorubicin are depicted in Fig. 3. Thonningiol (**9**), seputhecarpan D (**13**) as well as doxorubicin activated caspases 3/7 and 9 in MCF-7 cells, with optimal activities observed generally in cells treated with IC_50_. The increases were 3.97-fold, 4.66-fold and 2.02-fold for caspases 3/7 and 2.67-fold, 3.47-fold and 1.3-fold for caspase 9 respectively for compounds **9, 13** and doxorubicin.

**Fig. 4:**
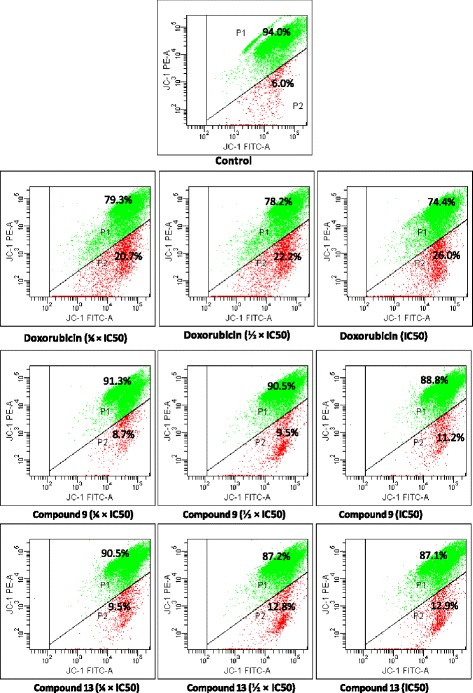
Effects of thonningiol (9), seputhecarpan D (13) and doxorubicin on MMP in MCF-7 cells for 72 h. IC_50_: 0.36 μM (thonningiol), 9.78 μM (seputhecarpan D) and 0.35 μM (doxorubicin). P1: cells with intact MMP; P2: cells with altered MMP

The effects of compounds **9**, **13** and doxorubicin in the integrity of MMP in MCF-7 cells are summarized in Fig. 4. Phytochemicals **9** (11.2%) and **13** (12.9%) slightly altered the MMP in MCF-7 cells, contrary to doxorubicin that induced up to 26.0% depletion at IC_50_.

**Fig. 5:**
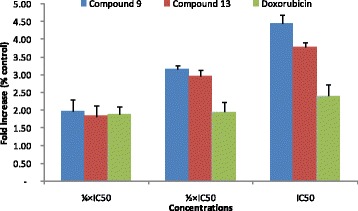
Production of ROS in MCF-7 cells treated with thonningiol (9), seputhecarpan D (13) for 24 h. IC_50_: 0.36 μM (thonningiol), 9.78 μM (seputhecarpan D) and 0.35 μM (doxorubicin)

The production of ROS in MCF-7 cells treated with compounds **9**, **13** and doxorubicin is shown in Fig. 5. Thonningiol (**9**), seputhecarpan D (**13**) and doxorubicin induced increase in ROS levels respectively by 4.46-fold, 3.78-fold and 2.40-fold at IC_50_ as compared to non-treated cells.

## Discussion

In the present investigation, we assessed the ability of 14 phytochemicals from African medicinal plants to prevent the proliferation of four cancer cell lines namely breast, colon, lung and liver cells. They are amongst the frequently diagnosed cancers globally [3]. The threshold recognized for a good phytochemical is the IC_50_ values around or below 4 μg/mL or 10 μM as defined by the National Cancer Institute (NCI) [8, 30, 31]. Amongst the 14 tested compounds, six (**3, 9–13**) had recordable IC_50_ values in all the four tested cancer cell lines.

To the best of our knowledge, the cytotoxicity of compounds **9** and **11** is being reported for the first time here. The triterpenoid16*β*-hydroxylupeol (**3**) is a hydroxylated derivative of the known cytotoxic compound, lupeol. In effect, the cytotoxicity of lupeol has been reported in several cell lines including colorectal cancer, gastric cancer or liver cancer cells [32–35]. The cytotoxicity of compound **3** is therefore in accordance with published literature and this work provides more information on the activity of lupeol derivatives. The cytotoxicity of compounds **10, 12** and **13** was reported against A549 and SPC-212 lung cancer cells [12]. The present work brings additional data on the cytotoxic potential of these compounds. In this study, IC_50_ values below 10 μM were obtained with compounds **9** and **13** in 4/4 and 3/4 tested cell lines respectively. This is an indication that these two molecules have promising cytotoxic potential.

Cysteine-aspartic proteases commonly known as caspases are protease enzymes essential for programmed cell death and inflammation [36]. In this study, it was found that phytochemicals **9, 13** as well as doxorubicin induce apoptotic cell death in MCF-7 cells with increase in caspases 3/7 and 9 activities (Figs. 2 and 3). These data indicate that activation of caspases is one of the modes of action of compounds **9** and **13**. Flavonol such as isorhamnetin was also shown to induce apoptosis in cancer cells by activation of caspases [37, 38], consolidating the results obtained with compound **9** belonging to the class of dihydroflavonol. However, isorhamnetin rather exerted cell cycle arrest in G2/M in HCT116 colon cancer cells contrary to compound **9** that exerted arrest in G0/G1 as observed in this study [38]. Also, pterocarpan isoflavonoids such as sophorapterocarpan A and isoneorautenol previously induced apoptosis in CCRF-CEM leukemia cells through activation of caspases 3/7, 8 and 9 as well as the loss of MMP and increased ROS production [15, 16]. In this study, it was also found that phytochemicals **9** and **13** slightly induced MMP alteration but enhanced ROS production in MCF-7 cells (Figs. 4 and 5). The activation of caspases 3/7 (effector caspases) and 9 (initiator caspases) (Fig. 3) as well as the low alteration of MMP, probably due to the low concentrations of compounds tested (¼ × IC_50_, ½ × IC_50_ and IC_50_), is an indication that intrinsic mitochondrial pathway could be involved in the cytotoxic effect of compounds **9** and **13** [39]. Mitochondria play a central role in cellular metabolism as main ATP source, and during ATP biosynthesis, ROS are generated. Mitochondria-targeting compounds kill cancer cells due to their ability to initiate mitochondrial outer membrane permeabilization [9, 40]. This also indicates that ROS production is another mode of apoptotic cell death induced these two phytochemicals.

Regarding the structure-activity relationship, it appears that the best spectra of activity were achieved with triterpenoid **3**, flavonol **9** and the 4 tested isoflavonoids **10–13**. Five terpenoids including one steroid (**1**) and four trierpenoids (**2–5**) were tested. The steroid **1** as well as triterpenoids **2**, **4** and **5** had low and selective cytotoxic effects (Table 1). Only triterpenoid **3** with two hydroxyl (-OH) group in C3 and C16 was active towards the four tested cancer cell lines. Hydroxylation of triterpenoids therefore seems to increase the cytotoxic effect. However, steroid **1** with only one –OH group in C3 had poor activity. Flavonoids tested herein included glycosylated flavonols **7** and **8** as well as non glycosylated flavonol **9**. Amongst them, compounds **7** and **8** had low and selective cytotoxicity, with a recordable IC_50_ values obtained only towards MCF-7 cells. In contrast, dihydroflavonol **9** had significant cytotoxic effect (IC_50_ values below 10 μM) in 4/4 tested cancer cell lines (Table 1). It can therefore be deduced that glycosylation of flavonoids in C3 significantly reduces their cytotoxic effect. The four tested isoflavonoids (**10–13**) were active in all cancer cell lines. Amongst them, pterocarpan **13** had the best activity with IC_50_ values below 10 μM against 3/4 cancer cells. Contrary to pterocarpans **11** and **12**, pterocarpan **13** do not have an additional furo- cycle; this is an indication that additional furo- cycle of pterocarpans reduces the degree of activity. Between pterocarpans **11** and **12**, the substitution of 4’-OH by 4’-OCH_3_ seems not to significantly influence their activity (Table 1). The only tested xanthone (**14**) displayed selective cytotoxicity. However, this effect was significant towards Caco-2 cells (IC_50_ value of 3.15 μM).

## Conclusions

The present study highlights the good potential of the African flora as a source of cytotoxic phytochemicals to combat malignant diseases. Thonningiol (**9**) and seputhecarpan D (**13**) are good antiproliferative molecules that should be explored more in future to develop a cytotoxic drug to fight human carcinoma. Dihydroflavonol **9** and pterocarpan **13** induced apoptosis in MCF-7 cells through activation activator caspase 9 and effector caspases 3/7 as well as enhanced ROS production.

## Additional file

Additional file 1: S1. Details on extraction and isolation of compounds (**1, 4, 6, 7, 8**) from *Synsepalum zenkeri*; S2. Further details on tested compounds. (DOCX 24 kb)

## Abbreviations

1: *β*-spinasterol
**10**: glyasperin F
**11**: seputhecarpan B
**12**: seputhecarpan C
**13**: seputhecarpan D
**14**: rheediaxanthone A
**2**: friedelanone
**3**: 16*β*-hydroxylupeol
**4**: *β*-amyrin acetate
**5**: 5-*O*-methyl-myo-inositol or sequoyitol
**6**: lupeol acetate
**7**: rhamnitrin or 7-*O*-methylquercetin 3-*O*-rhamnoside
**8**: europetin 3-*O*-rhamnoside or 7-*O*-methylmyricetin 3-*O*-rhamnoside
**9**: thonningiol
CH_2_Cl_2_: dichloromethane
CHCl_3_: chloroform
DMSO: dimethylsufoxide
EtOAc: ethyl acetate
H_2_DCFH-DA: 2′,7′-Dichlorodihydrofluorescein diacetate
MeOH: methanol
MMP: mitochondrial membrane potential
NR: neutral red
ROS: reactive oxygen species
TLC: thin layer chromatography
