# Supplementary material for: Cytotoxicity of seputhecarpan D, thonningiol and 12 other phytochemicals from African flora towards human carcinoma cells
Source: BMC Complement Altern Med. 2018 Jan 30;18:36. doi: 10.1186/s12906-018-2109-9 (PMC5789597; doi:10.1186/s12906-018-2109-9)
Supplement: Additional file 1: — S1. Details on extraction and isolation of compounds (1, 4, 6, 7, 8) from Synsepalum zenkeri; S2. Further details on tested compounds. (DOCX 24 kb) [file 12906_2018_2109_MOESM1_ESM.docx]

**Cytotoxicity of seputhecarpan D, thonningiol and 12 other phytochemicals from African flora towards human carcinoma cells**

Victor Kuete^1,2*^, Dominique Ngnintedo^3^ , Ghislain W. Fotso^3^, Oğuzhan Karaosmanoğlu^2,4^, Bonaventure T. Ngadjui^3,5^, Felix Keumedjio^3^, Samuel O. Yeboah^6^, Kerstin Andrae-Marobela^7^, and Hülya Sivas^2^

*^1^Department of Biochemistry, Faculty of Science, University of Dschang, Dschang, Cameroon;*

^2^*Department of Biology, Science Faculty, Anadolu University, Eskişehir, Turkey;*

*^3^Department of Organic Chemistry, Faculty of Science, University of Yaoundé I, Yaoundé, Cameroon,*

^4^*Department of Biology, KamilÖzdağ Science Faculty, KaramanoğluMehmetbey University, Karaman, Turkey;*

*^5^Department of Pharmacognosy and Pharmaceutical Sciences, Faculty of Medicine and Biomedical Science, University of Yaoundé I, Yaoundé, Cameroon*

*^6^Department of Chemistry, faculty of Science, University of Botswana, Block 237, Private Bag 0022, Gaborone, Botswana*

*^7^Department of Biological Sciences, Faculty of Science, University of Botswana, Block 235, Private Bag, 0022, Gaborone, Botswana*

*Correspondence:*

** Tel: +237 677355927; E-mail: Kuetevictor@yahoo.f (Victor Kuete)*

**Authors-emails:**

Victor Kuete: kuetevictor@yahoo.fr

Ghislain W. Fotso: ghis152001@gmail.com

Dominique Ngnintedo: blackynintendo@gmail.com

Oğuzhan Karaosmanoğlu: oguzhankaraosmanoglu@windowslive.com

Bonaventure T. Ngadjui: ngadjuibt@yahoo.fr

Samuel Yeboah: yeboahso@mopipi.ub.bw

Felix Keumedjio: keumedjiofelix@yahoo.fr

Kerstin Andrae-Marobela: marobelak@mopipi.ub.bw

Hülya Sivas: hzeytino@anadolu.edu.tr

**S1. Extraction and isolation of compounds from *Synsepalum zenkeri***

The air-dried powder of the leaves (1000 g), bark (2000 g) and roots (1500 g) of *Synsepalum zenkeri*, were extracted by maceration at room temperature with the mixture dichloromethane/methanol (CH_2_Cl_2_/MeOH 1:1). Crude extracts (126.52 g, 175.34 g and 138.62 g) respectively after removal of the solvent under reduced pressure, were suspended in water and partitioned successively with hexane, dichloromethane, ethyl acetate and *n*-butanol.

The hexane fraction of the bark (50 g) was absorbed on 70 g of silica and chromatographed over a silica gel column using hexane and the mixture of hexane-ethyl acetate (EtOAc) of increasing polarity. 168 fractions of 200 mL each were collected and combined according to their thin layer chromatography (TLC) profile to give 9 sub-fractions (F_1_-F_9_): F_1_ [(1-17), *n*-hexane], F_2_ [(18-35), *n*-hexane-EtOAc 2.5%], F_3_ [(36-58), *n*-hexane- EtOAc 5%], F_4_ [(59-83), *n*-hexane- EtOAc 7.5%], F_5_ [ (84-113), *n*-hexane- EtOAc 10%], F_6_ [(114-138), *n*-hexane-EtOAc 12.5%], F_7_ [(139-160), *n*-hexane-EtOAc 15%], F_8_ [(161-165), EtOAc] and F_9_ [(166-168), MeOH]. The mixture of F_1_-F_3_ precipitated, and the precipitate was washed with *n*-hexane to give 90 mg of *β*-amyrin acetate (**4**) [1] while F_5_ was chromatographed to give 13 mg of *β*-spinasterol (**1**) [2].

The dichloromethane (15 g) and EtOAc (18 g) fractions of the leaves of *S. zenkeri* were mixed together (33 g) according to their TLC profile and absorbed on 35 g of silica and chromatographed over a silica gel column with a mixture of chloroform (CHCl_3_)-MeOH of increasing polarity. 200 fractions of 200 mL were collected and combined according to their TLC profile to give 10 sub-fractions (F_1_-F_10_). F_1_ [(1-24), chloroform], F_2_ [(25-34),CHCl_3_-MeOH 2.5%], F_3_ [(35-53), CHCl_3_-MeOH 5%], F_4_ [(54-72), CHCl_3_-MeOH 7.5%], F_5_ [ (73-110), CHCl_3_-MeOH 10%], F_6_ [(111-149), CHCl_3_-MeOH 12.5%], F_7_ [(150-170), CHCl_3_-MeOH 15%], F_8_ [(171-190), CHCl_3_-MeOH 20%], F_9_ [(192-196), CHCl_3_-MeOH 25%] and F_10_ [(197-200), MeOH]. The mixture of F_4_-F_5_ was subjected to column chromatography to give a mixture of 52 mg of rhamnitrin or 7-*O*-methylquercetin 3-*O*-rhamnoside (**7**) [3] and 70 mg of europetin 3-*O*-rhamnoside or 7-*O*-methylmyricetin 3-*O*-rhamnoside (**8**) [3], that were separated through sephadex LH-20 eluted with mixture CHCl_3_-MeOH 30 %.

The dichloromethane fraction of the roots of *S. zenkeri* (25 g) was subjected to column chromatography over silica gel. Gradient elution was carried out using *n*-hexane and increasing the polarity with dichloromethane, EtOAc and MeOH. 140 fractions of 200 mL were collected. Monitoring by TLC, they were combined to give 14 sub-fractions (F_1_-F_10_). F_1_ [(1-15), *n*-hexane], F_2_ [(16-30), n-hexane-EtOAc 5%], F_3_ [(31-40), *n*-hexane-AcOEt 10%], F_4_ [(41-50), *n*-hexane-EtOAc 15%], F_5_ [ (51-60), *n*-hexane EtOAc 20%], F_6_ [(61-70), *n*-hexane- EtOAc 25%], F_7_ [(71-80), *n*-hexane-EtOAc 30%], F_8_ [(81-90), *n*-hexane-EtOAc 40%], F_9_ [(91-100), *n*-hexane-EtOAc 50%], F_10_ [(101-110), *n*-hexane-EtOAc 60%], F_11_[(111-120), *n*-hexane- EtOAc 70%], F_12_ [(121-130), *n*-hexane-EtOAc 80%], F_13_ [(131-135), EtOAc] and F_14_ [(136-140), MeOH]. The mixtures F_1_-F_3_ was subjected to column chromatography and eluted with the mixture *n*-hexane-AcOEt (90:10) and afforded lupeol acetate **6** (32 mg) [4].

*S2. Tested compounds.* *β*-spinasterol C_29_H_48_O (**1;** white powder; m/z: 412); friedelanone C_30_H_50_O (**2;** white powder; m/z: 426), 16*β*-hydroxylupeol C_30_H_50_O_2_ (**3;** white powder; m/z: 442), β-amyrin acetate C_32_H_52_O_2_ (**4;** white crystal; m/z: 468) and lupeol acetate C_32_H_52_O_2_ (**5;** white powder; m/z: 668), 5‑*O*‑methyl‑myo‑inositol or sequoyitol C_7_H_14_O_6_ (**6;** white powder; m/z: 194), rhamnitrin or 7-*O*-methylquercetin 3-*O*-rhamnoside C_22_H_22_O_11_ (**7;** yellow crystal; m/z: 462) and europetin 3-*O*-rhamnoside or 7-*O*-methylmyricetin 3-*O*-rhamnoside C_22_H_22_O_11_ (**8;** yellow crystal; m/z: 478) and thonningiol C_21_H_20_O_6_ (**9;** yellow oil; m/z: 368); glyasperin F C_20_H_16_O_6_ (**10;** yellow powder; m/z: 354), seputhecarpan B C_20_H_18_O_5_ (**11;** white powder; m/z: 338), seputhecarpan C C_21_H_20_O_5_ (**12;** brownish powder; m/z: 352), seputhecarpan D C_21_H_22_O_4_ (**13;** yellow oi**l** ; m/z: 338), rheediaxanthone A C_23_H_20_O_6_ (**14;** yellow powder; m/z: 392).

References

1. Okoye NN, Ajaghaku DL, Okeke HN, Ilodigwe EE, Nworu CS, Okoye FB. beta-Amyrin and alpha-amyrin acetate isolated from the stem bark of *Alstonia boonei* display profound anti-inflammatory activity. *Pharm Biol* 2014, 52(11):1478-1486.

2. Wandji J, Tillequin F, Mulholland DA, Wansi JD, Fomum TZ, Fuendjiep V, Libot F, Tsabang N. Fatty acid esters of triterpenoids and steroid glycosides from *Gambeya africana*. *Planta Med* 2002, 68(9):822-826.

3. Shen JC, Chien-Kuang C, Shoei-Sheng L. Polar constituents from Sageretiathea leaf characterized by HPLC-SPE-NMR assisted approaches. *J Chin Chem Soc* 2009, 56:1002-1009.

4. Rasoanaivo LH, Wadouachi A, Andriamampianina TT, Andriamalala SG, Razafindrakoto EJB, Raharisololalao A, Randimbivololona F. Triterpenes and steroids from the stem bark of *Gambey aboiviniana* Pierre. *J Pharmacogn Phytochem* 2014, 3:68-72.

5. Fotso GW, Ntumy AN, Ngachussi E, Duber M, Mapitse R, Kapche DGFW, Andrae-Marobela K, Ngadjui BT, Abegaz BM. Epunctanone, a new benzophenone, and further secondary metabolites from *Garcinia epunctata* Stapf (Guttiferae). *Acta Chim Helvet* 2014, 97:957-964.

6. Fotso GW, Maher FA, Ngnintedo D, Ango PY, Kapche DGFW, Ngameni B, Ngwenya B, Yeboah SO, Ngadjui BT, Andrae-Marobela K. Three new isoflavonoids with antioxidant properties from *Ptycholobium contortum* (N.E.Br.) Brummitt (Leguminosae). *Phytochemistry Letters* 2015, 14:254-259.

7. Ngnintedo D, Fotso GW, Kuete V, Nana F, Sandjo LP, Karaosmanoglu O, Sivas H, Keumedjio F, Kirsch G, Ngadjui BT *et al*. Two new pterocarpans and a new pyrone derivative with cytotoxic activities from *Ptycholobium contortum* (N.E.Br.) Brummitt (Leguminosae): revised NMR assignment of mundulea lactone. *Chem Cent J* 2016, 10:58.
